# Oxytocin in Women’s Health and Disease

**DOI:** 10.3389/fendo.2022.786271

**Published:** 2022-02-15

**Authors:** Ning Liu, Haipeng Yang, Liqun Han, Mingxing Ma

**Affiliations:** ^1^ Department of Obstetrics and Gynecology, Shengjing Hospital of China Medical University, Shenyang, China; ^2^ Neonatal Division of the Department of Pediatrics, The Fourth Affiliated Hospital of Harbin Medical University, Harbin, China; ^3^ Department of Radiology, The Fourth Affiliated Hospital of Harbin Medical University, Harbin, China; ^4^ Department of Colorectal Cancer Surgery, Shengjing Hospital of China Medical University, Shenyang, China; ^5^ Department of General Surgery, Shengjing Hospital of China Medical University, Shenyang, China

**Keywords:** lactation, menstruation, parturition, pregnancy, menopause

## Abstract

Oxytocin (OT) is a nonapeptide mainly produced in the supraoptic and paraventricular nuclei. OT in the brain and blood has extensive functions in both mental and physical activities. These functions are mediated by OT receptors (OTRs) that are distributed in a broad spectrum of tissues with dramatic sexual dimorphism. In both sexes, OT generally facilitates social cognition and behaviors, facilitates parental behavior and sexual activity and inhibits feeding and pain perception. However, there are significant differences in OT levels and distribution of OTRs in men from women. Thus, many OT functions in men are different from women, particularly in the reproduction. In men, the reproductive functions are relatively simple. In women, the reproductive functions involve menstrual cycle, pregnancy, parturition, lactation, and menopause. These functions make OT regulation of women’s health and disease a unique topic of physiological and pathological studies. In menstruation, pre-ovulatory increase in OT secretion in the hypothalamus and the ovary can promote the secretion of gonadotropin-releasing hormone and facilitate ovulation. During pregnancy, increased OT synthesis and preterm release endow OT system the ability to promote maternal behavior and lactation. In parturition, cervix expansion-elicited pulse OT secretion and uterine OT release accelerate the expelling of fetus and reduce postpartum hemorrhage. During lactation, intermittent pulsatile OT secretion is necessary for the milk-ejection reflex and maternal behavior. Disorders in OT secretion can account for maternal depression and hypogalactia. In menopause, the reduction of OT secretion accounts for many menopausal symptoms and diseases. These issues are reviewed in this work.

## 1 Introduction

Why should we concern women’s health and diseases? Women exhibit menstrual cycle, pregnancy, parturition, lactation, menopause and other unique physiological activities, such as maternal behaviors. Correspondingly, women have some unique reproduction-associated diseases, such as postpartum depression and menopausal syndromes. Women’s biological activities are regulated not only by the hypothalamic-pituitary-gonad (HPG) axis, but also by oxytocin (OT). While OT commonly influences sexual behaviors, production of sex steroids and the maturation of gemmates of both sexes (1), it differently influences women’s health and disease at different reproductive stages. In this work, we review the roles of OT in women’s health and disease.

## 2 General View of the OT System

OT, a classical neuropeptide, is mainly produced in hypothalamic OT neurons. Changes in OT neuronal activity can modulate cognitive, endocrine and physical activities as well as autonomic and visceral neural activities. In addition, scattered OT cells are also present in extrahypothalamic brain regions and peripheral sites, exerting autocrine and paracrine functions at local levels.

### 2.1 Histological Features of the OT System

OT neurons in the brain are largely aggregated in several neuroendocrine nuclei, typically the supraoptic nucleus (SON) and paraventricular nucleus (PVN). In the SON and PVN, most OT neurons send axons to the posterior pituitary and are magnocellular neuroendocrine cells. These OT neurons can release OT into the blood from OTergic terminals in the posterior pituitary and into the forebrain from OTergic axon collaterals (2). Another type of OT neurons in the CNS is parvocellular OT neurons. They are mainly present in the parvocellular division of the PVN and project to the brainstem and spinal cord but not the posterior pituitary (3). Alongside the hypothalamic OT neurons, some OT cells are also present in extrahypothalamic regions in the CNS and peripheral sites, such as amygdala, the median preoptic nucleus, uterus, placenta, amnion, corpus luteum, testis, heart and colon (4–6). Notably, OT gene expression in chorio-decidual tissues can increase three- to fourfold around the time of labor onset (7–10), supporting OT functional role in parturition. These histological features allow OT to modulate body functions at multiple levels and patterns including neuromodulation, neurosecretion, endocrine, autocrine and paracrine effects.

### 2.2 Features of OT Neuronal Secretion and Its Regulation

OT release from OT neurons is microdomain-specific (11). In response to changes in the neurochemical environment around OT neurons, changes in cytosolic Ca^2+^ level and/or firing activity occur. Increased firing rate causes OT release at the axonal terminals *via* excitation-secretion coupling. Increased cytosolic Ca^2+^ level triggers OT release from somata and dendrites. The somatodendritic OT release can be in synchrony with the firing activity under physiological conditions, such as suckling stimulation (12). It can be independent of the firing activity under some pathological conditions, such as maternal depression following offspring deprivation (13) and cesarean delivery (14).

**Figure 1:**
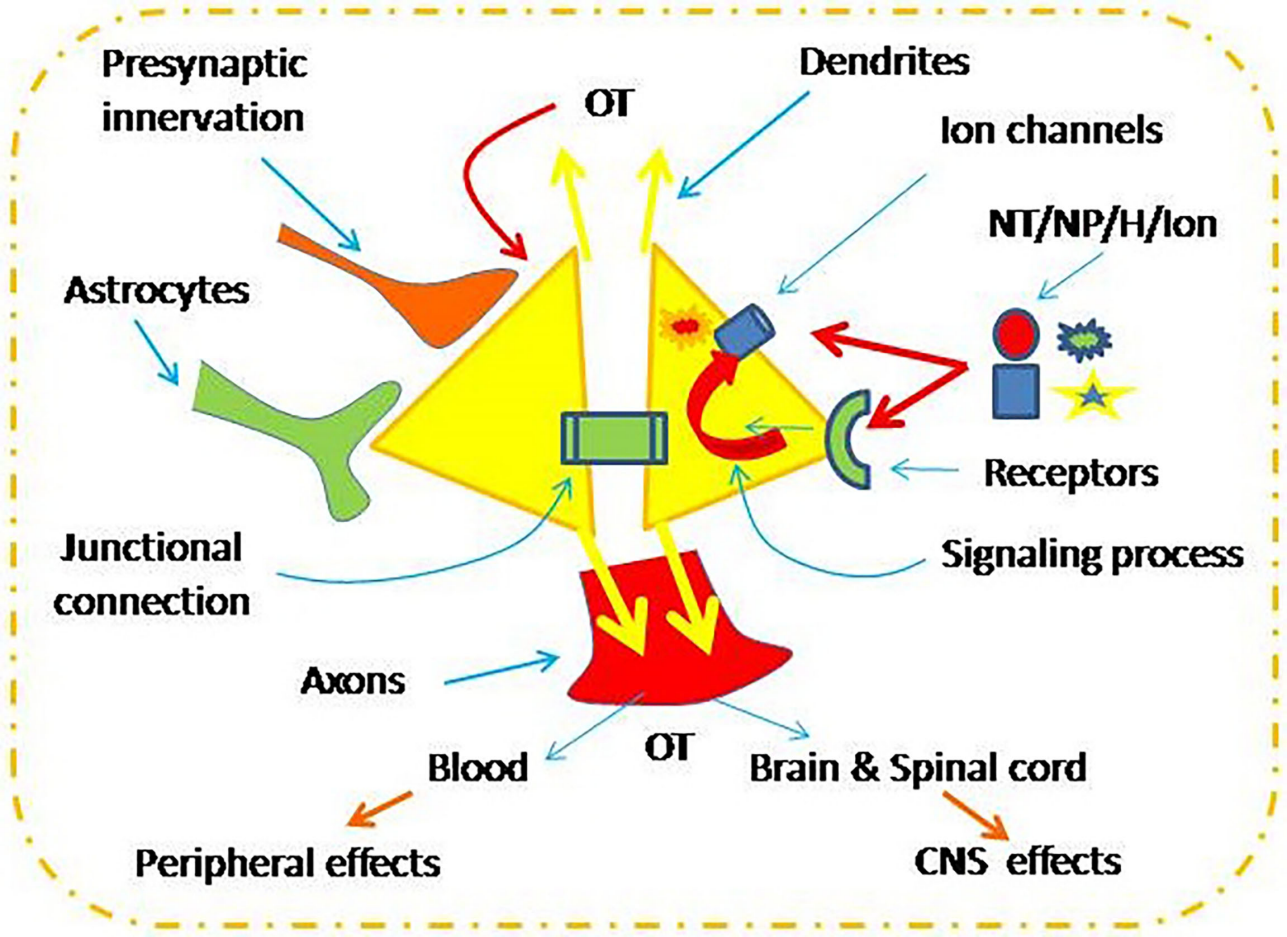
Neurochemical regulation of oxytocin (OT) neuronal activity. CNS, the central nervous system; NT/NP/H/Ion, neurotransmitters, neuropeptide, hormone and ions.

The electrical activity, cytosolic Ca^2+^ level and the resultant OT secretion are under the regulation of extracellular and intracellular factors (**Figure 1**). These factors include changes in neurochemical environment (15), tonic and clustered synaptic inputs (16, 17), intercellular junctional coupling such as connexin 36 (18), and astrocytic plasticity (19, 20). Moreover, autoregulation of OT neuronal activity is a key regulator of OT neuronal activity (21). By activation of OT receptors (OTRs), OT causes activation of a series of intracellular signaling events, such as increased cyclooxygenase-2 (21), extracellular signal-regulated protein kinases 1 and 2 (22), and protein kinase A (13). These signals can activate hyperpolarization-activated cyclic nucleotide-gated channel 3 (13, 23), which can promote OT secretion (24, 25). Under the regulation of these extracellular and intracellular factors, OT neuronal activity and OT secretion can meet the demands of body activity in response to environmental changes.

### 2.3 Expression of OTRs and Its Regulation

OTRs are the protein product encoded by OTR gene that is localized at 3p25-3p26.2 in the human chromosome (4). OTR belongs to the G-protein-coupled receptor superfamily and its activation is regulated by cholesterol as an allosteric modulator (26). The expression profile of OTRs is a tissue- and stage-specific, such as upregulation of the nuclear fractions in term myometrium and down-regulation in non-pregnant myometrium. The OTR gene appears to be highly methylated. Methylation around intron 1 and in intron 3 might contribute to tissue-specific suppression of the gene. OTRs are also regulated by the mechanisms of desensitization, which causes the loss of ligand-binding activity of the protein as well as suppression of OTR mRNA transcription. OTR mRNAs are present in different sizes, for instance 3.6 kb in human breast and 4.4 kb in ovary, endometrium, and myometrium. In posttranslational modifications, OTRs are further palmitoylated and glycosylated (27, 28). In addition, species differences are present and may be due to the existence of different clones of OTR genes of the myometrium and the hypothalamus at different reproductive stages (29, 30). Thus, the phenotype of OTRs from different species and different tissues of the same species could appear in different sizes.

In mammals, OTRs have been identified in a broad spectrum of tissues, including the kidney, heart, thymus, pancreas, adipocytes and other sites in addition to the CNS (4). Expressions of OTRs in the hypothalamus, uterus, and mammary glands are stimulated by estrogen (31). In females, OTRs are specifically localized in the myoepithelial cells of the mammary glands, and in the myometrium and endometrium of the uterus. Peripheral actions of OT are commonly associated with smooth muscle contraction, particularly within the female and male reproductive tracts (32).

OT expression during pregnancy and parturition in females has some unique features unseen in males. Changes in brain OT binding sites during pregnancy may influence the sudden onset of maternal behavior in female rats at parturition. Transcriptional regulation of OTR gene expression mediates changes in receptor density in the brain in a region-specific manner during pregnancy, such as the uterus (33), the PVN, SON, the bed nucleus of stria terminalis (BNST) and the medial preoptic area (mPOA) (34). Moreover, peak OTR mRNA expression was observed at parturition in the SON, brainstem regions, mPOA, BNST, and olfactory bulbs. Postpartum OTR expression in all brain regions returned to levels observed in virgin rats (35). These features of OTR expression are in agreement with the demands of establishing maternal behaviors and parturition.

Correspondingly, the uterus transitions from a quiescent non-contractile state to an active contractile state at the end of pregnancy. This is in association with increased OT/OTR signals (10, 36). Uterine quiescence requires prevention of excessive Ca^2+^ influx through voltage-dependent Ca^2+^channels at the human myometrial smooth muscle cells. Through most time of pregnancy, the K^+^ leak current is dominant and maintains the cell at a sufficiently negative membrane potential to prevent premature uterine contraction. However, increased myometrial OT/OTR expression near the term (10, 36) significantly increases OTR-associated Gαq-protein activation of protein kinase C (33), which then inhibits the activity of sodium-activated, high-conductance, K^+^ leak channel. This results in depolarization of the uterine smooth muscle cells and calcium entry that causes uterine contraction (37). This effect is associated with the induction of cyclooxygenase 2, production of prostaglandin F2α and connexin 43 of the uterus (38, 39).

## 3 OT Functions and Sexual Dimorphism

### 3.1 General Functions

The functions of OT depend on the distribution of OTRs and relative change in OT levels. In the brain and spinal cord, activation of OTRs is associated with a variety mental activities, such as social memory, pair-bonding, maternal behavior, and aggression and instinctive behaviors such as sexual activity, anxiety, feeding and pain perception (40–42). In the circulation, OT can facilitate parturition and the milk-ejection reflex and regulate activities of other organ systems (43, 44). Locally-produced OT can promote the differentiation of thymic cells, inhibit inflammation (45), protect the heart from ischemic injury (46), and suppress metastasis of colorectal cancer (5) among many other functions (1, 4).

In the CNS sites, OT can regulate social activity, instinctive behaviors, and visceral functions. For example, by activation of inhibitory neurotransmission in the medial frontal cortex, the amygdala and hippocampus, OT can promote social recognition and pro-social behaviors while reducing stress and fear (47–49). Released from parvocellular OT neurons in the PVN, OT can act on the ventral tegmental area (VTA) to activate rewarding process. OT can suppress nociception ad pain by acting on the periaqueductal gray and spinal cord. By acting on the dorsal vagal complex, OT can regulate visceral activities *via* vagus. By innervating median eminence and median preoptic area, OT can increase gonadotropin-releasing hormone (GnRH) release and the activity of HPG axis (1, 50). In addition, intrahypothalamic OT can inhibit corticotrophin-releasing hormone neurons and social stress *via* acting on the parvocellular division of the PVN (51).

By contrast, circulating and locally-produced OT can influence body functions at cellular, tissue, organ and system levels. For instance, OT can promote insulin secretion (52) and immunological homeostasis (53), protects cardiovascular system (46), suppresses colorectal cancer migration (5), and potentially antagonizes COVID-19 pathogenesis (54). In addition, OT is a natriuretic hormone that acts directly on the kidneys (55). On the other hand, its synthesis and secretion are regulated by changes in plasma osmolality and blood volume (56, 57). This process proceeds in an estrogen-dependent manner in females (58, 59). Thus, OT extensively modulates body functions.

### 3.2 Sexual Dimorphism of OT Functions

In studies on OT functions, significant difference between males and females emerges, which is particularly significant in psychological and reproductive functions.

#### 3.2.1 OT/OTR Signaling

Sexual dimorphism of OT functions is based on expression levels of OT and OTRs. For instance, serum OT levels are significantly higher among women than men (60), which makes women more sensitive to OT level reduction and likely accounts for menstrual pain (61) and higher incidence of depression in women (62). By contrast, OT binding sites in the VMH and dorsal horns are significantly high in males relative to females (63), which may contribute to the central regulatory actions of OT on feeding, reproduction *via* VMH (64) and nociception *via* the spinal cord (65). Males also exhibit higher OTR levels in the medial amygdala irrelevant to the reproductive status (66), which likely makes men less fearful facing stressful challenges because OT acing on the medial amygdala inhibits fear. Higher OTR levels in the nucleus accumbens are present at breeding males but not breeding females (66), which makes paternal behaviors conditional (67) and more rewarding (68). The sex dimorphism in the distribution OT and OTRs sets a histological basis for gender-specific functions and behaviors.

#### 3.2.2 Psychological Functions

The most relevant OT-associated sexual dimorphism is present in empathy, social skills and other higher brain functions. OT facilitates familiar-partner preference with females being more significant and it increases trust in others and reduces anxious emotion, especially for males (69). Autism spectrum disorder is relatively low in the female gender. This is related to the higher levels of OT and better pragmatic language in girls than boys (70). In nulliparous women, OT enhances attention to the baby schema and morphological characteristics of an infant’s face (71). In animal studies, the sexual dimorphisms of OT psychological functions are also identified. For example, in prairie vole, compared to females, males with less OTR expression perform better than females in a spatial memory and spatial learning test (72). Intranasal administration of OT (IAO) before the acquisition or recall sessions enhances conditioned safety memory in female rats while OT has no effects in male rats (73). In male prairie voles exposure to IAO during the peri-adolescent period impairs adult pair bonding in a dose-dependent fashion while IAO appears to facilitate pair bonding in females. This is associated with that IAO in females but not in males increases OTR binding in the nucleus accumbens shell (74). Thus, differences in the OT/OTR signaling determine sex dimorphisms between males and females.

#### 3.2.3 Peripheral OT Effects

In association with hypothalamic OT neuronal activity and OT secretion, OT can extensively modulate body activities at peripheral sites. In the rat with left ventricle infarction, OTR is down-regulated in females while up-regulated in males. Thus, OTR signaling and OT protection are suppressed stronger in ischemic myocardium in females than males. It also accounts for why females have higher risk of heart failure and death following myocardial infarction relative to males (75). Moreover, OT is involved in bone formation in both sexes during development; however, OT treatment has no effect on male osteoporosis because estrogen amplifies OT local autocrine and paracrine secretion (76). Decreased OT and increased OTR occur in male but not female alcohol- dependent rats and patients (77). Thus, differences in OT/OTR signaling also exist in the sex dimorphism of circulation and peripheral OT functions.

#### 3.2.4 Male Reproductive Functions

Among all OT functions, the most dramatic sexual dimorphism is reproductive functions. Relative to the periodic changes in females’ reproductive physiology, males do not have dramatic monthly periodic alteration in reproductive endocrine activity and reproductive functions. It is known that plasma OT levels increase during sexual arousal and are significantly higher during orgasm/ejaculation in both women and men (78). In the CNS levels, OT from the PVN in the VTA of rats induces penile erection by activating OT neurons-spinal cord pathway (79). This pathway involves activation of dopamine, glutamate and other neurons in the VTA that project to nucleus accumbens, prefrontal cortex, amygdala, and other forebrain regions (80). Moreover, this OT-PVN-VTA-forebrain pathways play a role in the motivational and rewarding aspects of sexual behavior (81). At peripheral sites, OT directly targets the erectile tissues including corpus spongiosum and corpus cavernosum, and promotes ejaculation by increasing sperm number and contracting ejaculatory tissues (82). Thus, OT is a pivotal regulator of male reproductive functions.

### 3.3 Maternal Behavior

Maternal behavior begins before breastfeeding near the term and is clearly associated with the actions of OT. For example, in OT knockout mice, maternal behavior is disrupted (83) and maternal behavior is reduced significantly when OTR signaling is blocked (84). By contrast, IAO rapidly increases maternal care in mice (85). It is believed that OT neurons in the PVN are sensitive for the smell of offspring in lactating and multiple-parturient rats (86). OT neurons in the PVN and SON project to the hippocampus, amygdala, ventral striatum, hypothalamus, nucleus accumbens and brainstem nuclei; they can extensively modulate maternal behaviors (2, 3). By modulation of the maternal behaviors, OT convincingly promotes human development as recently reviewed (87, 88).

Notably, women with peripartum exposure to synthetic OT have a higher relative risk of depressive or anxiety disorder diagnosis or antidepressant/anxiolytic prescription within the first year postpartum than women without synthetic OT exposure (89). This is likely due to the suppression of excessive OT on OT neuronal activity *via* a mechanism of post-excitation inhibition of OT neuronal activity (14, 23, 90).

### 3.4 OT and Diseases

Under pathological conditions, reduced OT release or disorders in OTR signaling can cause many diseases, such as social stress (51), schizophrenia and depression (91), obesity (92), lactation failure, postpartum depression (13, 13) and even mammary tumor (93). For instance, schizophrenia is a form of mental disorders and involves dysregulation of the OT system. From animal models to human studies, observations have revealed that OT can improve the psychopathology of patients with schizophrenia by regulating social cognition and behavior (94). Postpartum depression is associated with disorders on maternal OT system. As reported in rats, lack of suckling stimulation and bolus OT release during lactation can decrease maternal blood OT levels and cause milk insufficiency and maternal depression in lactating mothers deprived of offspring (13, 95) or following cesarean delivery (14). However, disrupted maternal behavior and lactation performance can be largely improved by IAO during offspring deprivation (13) or following cesarean delivery (14). Obviously, disorder in OT system activity contributes to disorders in mental activity while IAO has the potential to correct abnormal social behaviors such as those in schizophrenia and postpartum depression.

Notably, in mediating OT actions, the efficiency of OTRs depends on their gene polymorphisms, expression levels and posttranslational modification (96–98). For example, the GG homozygotes of OTR rs2254298 are associated with childhood adversity (99); DNA methylation in the 1st intron of the OTR gene causes common learning and behavioral impairments (100). Thus, OT-associated diseases are not only associated with disorders in OT secretion but also with abnormality of OT/OTR signaling.

## 4 OT Functions at Different Reproductive Stages of Women

### 4.1 Menstrual Cycle

It is well established that a menstrual cycle is regulated by HPG axis. However, studies also highlight a pivotal role of OT in menstrual cycle. For instance, increased brain OT level can increase GnRH secretion, specifically at pre-ovulation stage (101, 102). Consistently, plasma OT is significantly higher during ovulatory phases than the luteal phase in ovulating women (103). In rats, c-Fos expression in the SON is significantly higher and OT neurons-associated astrocytic processes retract significantly during the proestrus (104, 105). These findings support functional activation of OT neurons and increased OT release before ovulation. Thus, the stimulatory effect of OT on GnRH secretion can promote luteinizing hormone (LH) secretion, facilitate ovulation and prepare uterus environment for pregnancy.

**Figure 2:**
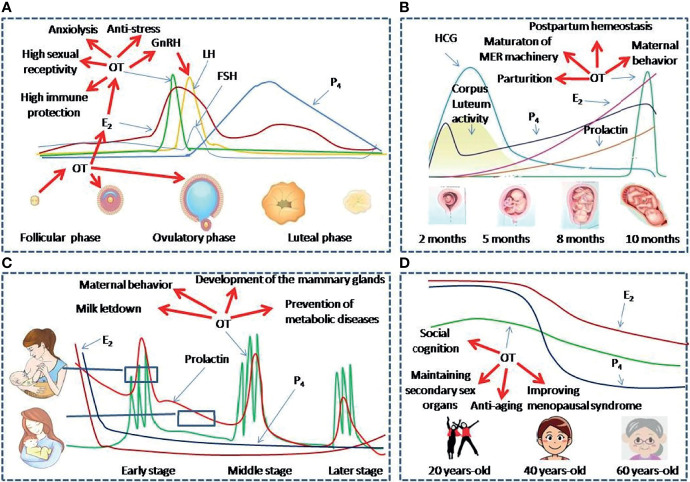
OT functions at different reproductive stages of women. **(A–D)** Panels show levels and functions of OT and associated reproductive hormones during Menstruation **(A)**, Pregnancy and parturition **(B)**, Lactation **(C)** and Peri-menopausal stage **(D)**, respectively. E2, estradiol; FSH, follicle-stimulating hormone; GnRH, gonadotropin-releasing hormone; HCG, Human Chorionic Gonadotropin; LH, Luteinizing hormone; MER, the milk-ejection reflex; P4, progesterone.

By contrast, exogenous OT can shorten postpartum estrous interval but triggering a delay in ovulation while a higher dose of OT could stimulate the growth of small, medium, and total follicles in postpartum buffaloes (106). Estradiol level increase is correlated with OT release from the pituitary and causes more luteal OT secretion (107). Thus, OT secretion and estrogen release before luteal phase can form a positive feedback loop and they together facilitate ovulation (**Figure 2A**).

During pregnancy, persistent release of uterine progesterone and estrogen interrupts the periodical activity of the HPG axis. Moreover, increased progesterone also inhibits OT release (108). They together cause the cessation of menstrual cycles. Following parturition, breastfeeding delays the resumption of normal ovarian cycles by disrupting the pattern of pulsatile release of GnRH from the hypothalamus. Intermittent bolus release of OT during suckling, its disruption of normal interaction of OT with the HPG axis and inhibition of energy intake also play a key role in lactation-associated amenorrhea (92). When suckling stimulus declines near weanling, preovulatory LH surge restores and ovulation takes place with the formation of a corpus luteum of variable normality.

By contrast, women with a history of primary or secondary dysmenorrhea have lower blood OT concentrations during menses, which is associated with worse menstrual pain and pain-related behavior (61). In women with early life sexual abuse, OT can positively modulate menstruation-related mood disorders (109). In addition, OT can enhance conditioned safety memory (73), elicit maternal behavior towards alien pups in virgin females (110), alleviate chronic pelvic pain (111) and rehabilitate anorexia nervosa (112). Thus, OT extensively modulates mental and physical activities and menstruation-associated diseases.

### 4.2 Pregnancy and Parturition

#### 4.2.1 OT and Pregnancy

During pregnancy as marked by the production of human chorionic gonadotropin, OT production increases gradually and prepares for the demands of parturition and breastfeeding (**Figure 2B**). As reported, gestation gradually increases OT synthesis and OTR expressions in magnocellular OT neurons in the SON and PVN and in forebrain neurons, such as the mPOA (34). However, OT release from OT neurons does not increase during pregnancy until the time shortly before parturition. This is clearly beneficial for avoiding abortion during pregnancy. By contrast, blockade of OTR during mid-late gestation delays OT release and causes hypogalactia during lactation (113). Thus, the development of OT/OTR signaling is an adaptive response for maintaining the safety of pregnancy. However, increased OT synthesis and preterm OT release in the hypothalamus are necessary for the maturation of hypothalamic machinery that allows OT to be released in bolus intermittently during parturition and lactation (113). Thus, OT actions during pregnancy highly match peri-partum physiological demands.

#### 4.2.2 OT and Parturition

Shortly before the parturition, progesterone inhibition of OT neuronal excitability *via* endogenous opioids and GABA is weakened (108), which allows OT release in bolus in response to extension of cervix. The mechanical stretch of the cervix can activate magnocellular OT neurons in burst-like firing activity, which determines a bolus release of OT from the posterior pituitary (114). OT further initiates a self-sustaining cycle of uterine contractions until fetus is expelled. In this process, uterine OT release is also increased, which intensifies circulating OT-evoked uterine contraction by increasing prostaglandin F production. As a result, parturition is accelerated (115) and postpartum hemorrhage is reduced (116). Thus, OT is an essential factor for natural delivery.

Notably, nocturnal and pulsatile OT release often occurs at the end of parturition. For example, plasma OT and nocturnal uterine activity in the dams increase progressively during late pregnancy and delivery in rhesus monkeys (117). This is associated with the effect of light/darkness on the pulsatile OT release (118), which determines the high incidence of parturition during night.

### 4.3 Lactation

In all mammals, OT is a hormone necessary for mothers to nurse their offspring through the milk-ejection reflex. Clearly, in OT-knockout mice, the pups cannot obtain milk from the mother because the dams fail to eject milk for the offspring to obtain through suckling the nipples (119). Consistently, conditioned OTR knockout dams experience high rate of pup mortality (120).

Successful breastfeeding depends on coordinated activities of numerous humoral factors. For instance, the increased hypothalamic OT release during suckling promotes prolactin secretion from the anterior pituitary. They together make milk production and ejection available for the baby during breastfeeding (121). This effect is different from the increased prolactin during pregnancy that acts to maintain the corpus luteum of the ovary and helps to sustain pregnancy but has no direct association with OT release (122). Under physiological condition and normal nutrition, breastfeeding in normally developed women relies on the milk-ejection reflex. In this reflex, suckling stimulation at the nipples activates OT neurons, causes OT release in a bolus into the blood, and results in the ejection of milk from the teat (**Figure 2C**). The activation of OT neurons appears as intermittently recurrent and simultaneous increase in the firing rate in a large pool of OT neurons over several seconds, which causes the bolus release of OT at OTergic neural terminals in the posterior pituitary (44). Without this synchronized burst firing of OT neurons, OT release from one or a few of OT neurons or only one side of the hypothalamus cannot trigger full milk ejections (123, 124). In the synchronized activation of OT neurons, simultaneous retraction of astrocytic processes from OT neurons, shared synapses, increased gap junctional coupling and cellular apposition are logically important contributors (44). However, for coordinated inter-nuclear burst synchrony among OT neurons, a synchronized signal from the mammillary body complex in the ventroposterior hypothalamus is the most important event. This is because disruption of it but not other brain regions disrupts inter-nuclear burst synchrony (125, 126).

Under the drive of exogenous factors including synaptic input, astrocytic plasticity and interneuronal interactions (**Figure 1**), a series of OTR-associated signaling events are activated during suckling. These events include mobilization of βɣ subunits (16), induction of cyclooxygenase 2 and prostaglandin production (21), phosphorylation of extracellular signal-regulated protein kinase 1 and 2 (22), activation of protein kinase A (13, 127), and filamentous actin reorganization (21, 22) among many others (44). These factors express in a different spatiotemporal order to modulate ion channel activity on the membrane (128), such as hyperpolarization-activated cyclic nucleotide-gated channel 3 (13, 23), and thus trigger burst firing in OT neurons and OT release. Nevertheless, detailed neural circuit between OT neurons and their regulatory network and the cellular mechanisms underlying burst firing remain to be explored.

About lactation-associated health issue, it has been extensively accepted that normal breastfeeding can reduce the incidence of postpartum depression, maternal obesity, diabetes, and even breast cancer. OT is necessary for these benefits of breastfeeding. As revealed in animal study, maternal behavior in OT knockout mice is incomplete (83). Lack of suckling stimulation and bolus OT release during lactation can decrease maternal blood OT levels and cause hypogalactia and maternal depression (13, 95). By contrast, IAO can largely restore maternal behavior and lactation performance as stated above. Importantly, OT can also suppress breast cancer. This is because OT can reduce the oxidative stress of the mammary glands and the occurrence of pre-cancer lesions in the mammary glands (129). Moreover, OT can down-regulate the NF-κB and up-regulation of miR-195. These molecules can promote cell apoptosis, inhibit cell proliferation and consequently, decrease the mammary tumor volume and weight (130). Thus, OT is not only essential for normal breastfeeding but also for the long-term health benefits of women.

### 4.4 Menopause

Menopause is the end of women’s menstrual cycles following decrease of reproductive hormones. From the middle age, blood OT levels decrease gradually (131). Decreased levels of OT can largely account for diminished sexual ability and vagal activity and reduced estrogen levels (**Figure 2D**).

#### 4.4.1 Sex Organs and Bone Metabolism

Menopausal atrophy of accessory reproductive organs is a common sign of reduced ovary functions. However, topical OT application can reverse vaginal atrophy (132, 133). Similarly, a high proportion of women develop osteoporosis after menopause, which increases the incidence of bone fractures. In animals, ovariectomy elicits bone loss and increased bone marrow adiposity (134). Administration of OT can normalize ovariectomy-induced osteopenia in mice by restoring osteoblast/osteoclast cross talk *via* the receptor activator of nuclear factor-κB ligand/osteoprotegerin axis (135). Thus, OT is a potential treatment of menopausal osteoporosis.

#### 4.4.2 Body Mass

Weight gain in menopausal women has been frequently reported (136). Obesity and menopause are independent negative predictors of plasma OT levels (131). Daily administration of OT can normalize body weight and intraabdominal fat depots in ovariectomized mice. This effect is mediated by inhibition of adipocyte precursor’s differentiation with a tendency to lower adipocyte size by shifting fuel utilization favoring lipid oxidation (135). In peri- and postmenopausal female rats, intraperitoneally administering OT also reduces serum triglyceride and low-density lipoprotein-cholesterol levels in naturally premenopausal or menopausal rats (137). Thus, OT can be a preventive factor of postmenopausal obesity, diabetes and the associated cardiovascular diseases (46).

#### 4.4.3 Cardiovascular Effects

Cardiovascular diseases increase dramatically in postmenopausal women. This is also associated with reduction of ovary functions and its influence on OT secretion. When estrogen production decreases, its activation of estrogen receptors on preautonomic PVN OT neurons is also weakened. Resultantly, OT regulation of HPG axis and baroreflex is weakened. This has been shown in ovariectomized rats (138). By contrast, OT can protect the cardiovascular system by maintaining cardiovascular integrity, suppressing atherosclerotic alterations and coronary artery disease, inhibiting metabolic disorders, inflammation and apoptosis and promoting regeneration and repair injuries (46).

As a whole, reduced OT secretion during aging can causes cardiovascular disease, osteoporosis, urinary incontinence, sexual malfunction, obesity, low metabolism after menopause. Thus, improvement of OT secretion can be an important strategy of anti-aging in women.

## 5 Conclusion

OT system can extensively modulate women’s physiology, particularly the cognitive and reproductive functions. While OT has common effects on the mental and physical activities of both men and women, different OT levels and OTR expressions at different reproductive stages regulate women’s reproductive activities differently. The influence of OT on women’s health mainly manifests as its modulation of menstrual cycle, pregnancy, production, lactation and menopause. Under pathological conditions, abnormality in OT secretion and/or OTR expressions can cause a series of female-specific diseases. Thus, further study on OT involvement in women’s health and disease is warranted. These studies should focus on women-specific mental and physical effects of OT and the underlying mechanisms, particularly during different stages of reproduction.

## Author Contributions

NL and MM: Conceived the study, wrote, revised and edited the text. HY and LH: Discussed the key contents, revised text and drew the figures. All authors contributed to the article and approved the submitted version.

## Conflict of Interest

The authors declare that the research was conducted in the absence of any commercial or financial relationships that could be construed as a potential conflict of interest.

## Publisher’s Note

All claims expressed in this article are solely those of the authors and do not necessarily represent those of their affiliated organizations, or those of the publisher, the editors and the reviewers. Any product that may be evaluated in this article, or claim that may be made by its manufacturer, is not guaranteed or endorsed by the publisher.
